# Detecting cognitive traits and occupational proficiency using EEG and statistical inference

**DOI:** 10.1038/s41598-024-55163-w

**Published:** 2024-03-07

**Authors:** Ilya Mikheev, Helen Steiner, Olga Martynova

**Affiliations:** 1 Department of Psychology, HSE University, Moscow, 101000 Russia; 2 Institute of Higher Nervous Activity and Neurophysiology of the Russian Academy of Sciences, Moscow, 117485 Russia; 3 Centre for Cognition and Decision Making, HSE University, Moscow, 101000 Russia

**Keywords:** EEG, Machine learning, Cognitive traits, Personality, Network models, Personality, Network models

## Abstract

Machine learning (ML) is widely used in classification tasks aimed at detecting various cognitive states or neurological diseases using noninvasive electroencephalogram (EEG) time series. However, successfully detecting specific cognitive skills in a healthy population, independent of subject, remains challenging. This study compared the subject-independent classification performance of three different pipelines: supervised and Riemann projections with logistic regression and handcrafted power spectral features with light gradient boosting machine (LightGBM). 128-channel EEGs were recorded from 26 healthy volunteers while they solved arithmetic, logical, and verbal tasks. The participants were divided into two groups based on their higher education and occupation: specialists in mathematics and humanities. The balanced accuracy of the education type was significantly above chance for all pipelines: 0.84–0.89, 0.85–0.88, and 0.86–0.88 for each type of task, respectively. All three pipelines allowed us to distinguish mathematical proficiency based on learning experience with different trade-offs between performance and explainability. Our results suggest that ML approaches could also be effective for recognizing individual cognitive traits using EEG.

## Introduction

Currently, there is no doubt about the specific association between different cognitive performances and electric patterns of brain activity, which can be recorded using electroencephalography (EEG)^1–5^. A relationship between EEG and cognitive operations can also be distinguished by statistical inference using machine learning models^6,7^. The resulting models are applicable for creating passive brain-computer interfaces (BCIs) that can adapt to a person’s cognitive abilities and educational background^8–11^.

Neuroscience utilizes machine learning for solving a wide spectrum of problems, such as the recognition of emotional and cognitive states, speech, psychiatric and neurodevelopmental disorders, and imaginary movements for the development of active BCIs^12–15^. The main challenge in applying classification methods to specific task-related EEG data is enhancing subject-independent model performance. The non-stationarity of EEG time series and the distortion of signals of interest in the presence of noise lead to high variability between trials recorded even from the same person and make training robust models a difficult task^16,17^. One of the most promising approaches is statistical approximation^18^ of EEG with projection to the new feature space where classes will have maximal separability.

However, the field of neuroscience has only recently started to focus on the phenomenon of mathematical giftedness^19,20^. While there have been numerous studies dedicated to the examination of mathematical abilities and the processes of solving mathematical problems, particularly arithmetic and spatial tasks, they have primarily focused on development mathematical skills in children and adolescents^21,22^.

Despite the considerable interest in studying mathematical abilities, the influence of specialized mathematical experience and education on the functional organization of the brain remains a relatively unexplored area in cognitive science. Only one study, published in 2016, addressed neuronal differences between mathematics and humanities specialists with comparable intelligence levels based on the brain functional magnetic resonance imaging^23^.

While there is a lack of data regarding the application of machine learning methods to recognize patterns of rhythmic EEG activity associated with complex cognitive tasks, this gap in knowledge is likely attributed to the challenges in interpreting the influence of individual brain areas and class separation^24^. On the other hand, the use of EEG to identify not only individual but also group differences in brain oscillatory activity is crucial for clinical applications^25^. Models trained on cross-subject classification tasks often exhibit low performance metrics and lack of generalizability^26^. Successful classification is typically associated with significant differences between classes. The very recent study^27^ applied machine learning analyses on high-density EEG data to classify math experts and novices, however the best accuracy was about 66 %.

Due to the linearity of Maxwell’s equations, EEG data are generated by a linear combination of brain sources with distortion by physiological and environmental noise. Thus, one of the most suitable representations of EEG for statistical inference and, in turn, solving classification problems is the spatial covariance matrix that, when noise is Gaussian, fully characterizes brain signals^28^.

Substantial information of EEG lies in the spatial domain of bioelectrical signal sources since the brain networks involved in different cognitive operations have distinct topography. Spatial filtering methods try to collect this information and discard the irrelevant ones. In the space of Symmetric Positive Definite (SPD) matrices, one can apply Common Spatial Pattern (CSP)^29^. Briefly, this method amounts to maximizing the variance of the (spatially) filtered signal under one condition while minimizing it for the opposite condition. Moreover, EEG spatial patterns provide a better understanding of neurophysiological processes underpinning brain oscillatory activity. Thus, backward models can ultimately be interpreted^30^.

In this study, we examine possible differences in cognitive traits between experts in mathematics (M) and in humanities (H), using EEG recorded during the solving of three types of tasks: arithmetic, logical, and verbal. The specialization is determined based on the area of higher education and working experience in the respective fields. We employ machine learning and features of oscillatory brain activity to classify individual personality traits associated with the specificity of education.

EEG research demonstrates a reproducible correlation of spectral power features of EEG with cognitive characteristics aligning with the ability of ensembles (Random Forest, Gradient Boosting) to learn effectively on non-linear but physiologically interpretable features. This motivated us to use spectral power features in the current study. We compared the robustness of three different pipelines: a linear model with supervised projection and log-diagonal vectorization (log-variance of extracted signals); a linear model with projection to a common space and estimation of geometric distance; and LightGBM with handcrafted non-linear power features. The advantage of gradient boosting methods (GBM) and, in particular, LightGBM^31^, is that during training, they minimize bias at the cost of being prone to overfitting. With correct preprocessing, noise whitening, and accurate parameter tuning, LightGBM can achieve state-of-the-art performance.To improve the overall generalizability of the classification, the number of components of projection methods and hyperparameters of machine learning models were tuned on a validation sample with a new set of subject EEG data.

Using linear patterns^30^ and SHapley Additive exPlanations^32^ values for explanation of features significance, we examined the difference in channels-frequency space between M and H groups during the solving of arithmetic, logical, and verbal tasks. Our results suggest that a machine learning approach could be effective for recognizing individual cognitive traits using EEG.

## Results

### Behavioral performance

The gender, age, and working memory capacity did not show significant differences between the M and H groups: z = − 1.93, p = 0.095; z = 1.29, p = 0.19; z = 0.02, p = 0.98 respectively. Moreover, there was no significant difference in hit ratio between the two groups. Reaction time (RT) varied depending on the task type for all participants, without a group effect: F(2,44) = 69.65, p < 1e−6. The longest RT was observed for arithmetical (Ar) tasks, the shortest for verbal (Verb) tasks with arithmetical sequence (Sq) task RT in the middle. The averaged RT for all three types of tasks showed significant differences in pairwise comparisons (p = 1e−4). However, the correctness (Hit) rate did not show any significant variations between the task types.

Furthermore, we examined the mean value of 96-channel data for each frequency range in correlation with behavioral results. After correcting for the multiplicity of comparisons (p < 0.007), a positive correlation (r = 0.53) emerged between PSD of the theta band and Hit AR rate, without significant influence of the group factor.

### Group differences in power spectral density of EEG during task-solving

**Figure 1:**
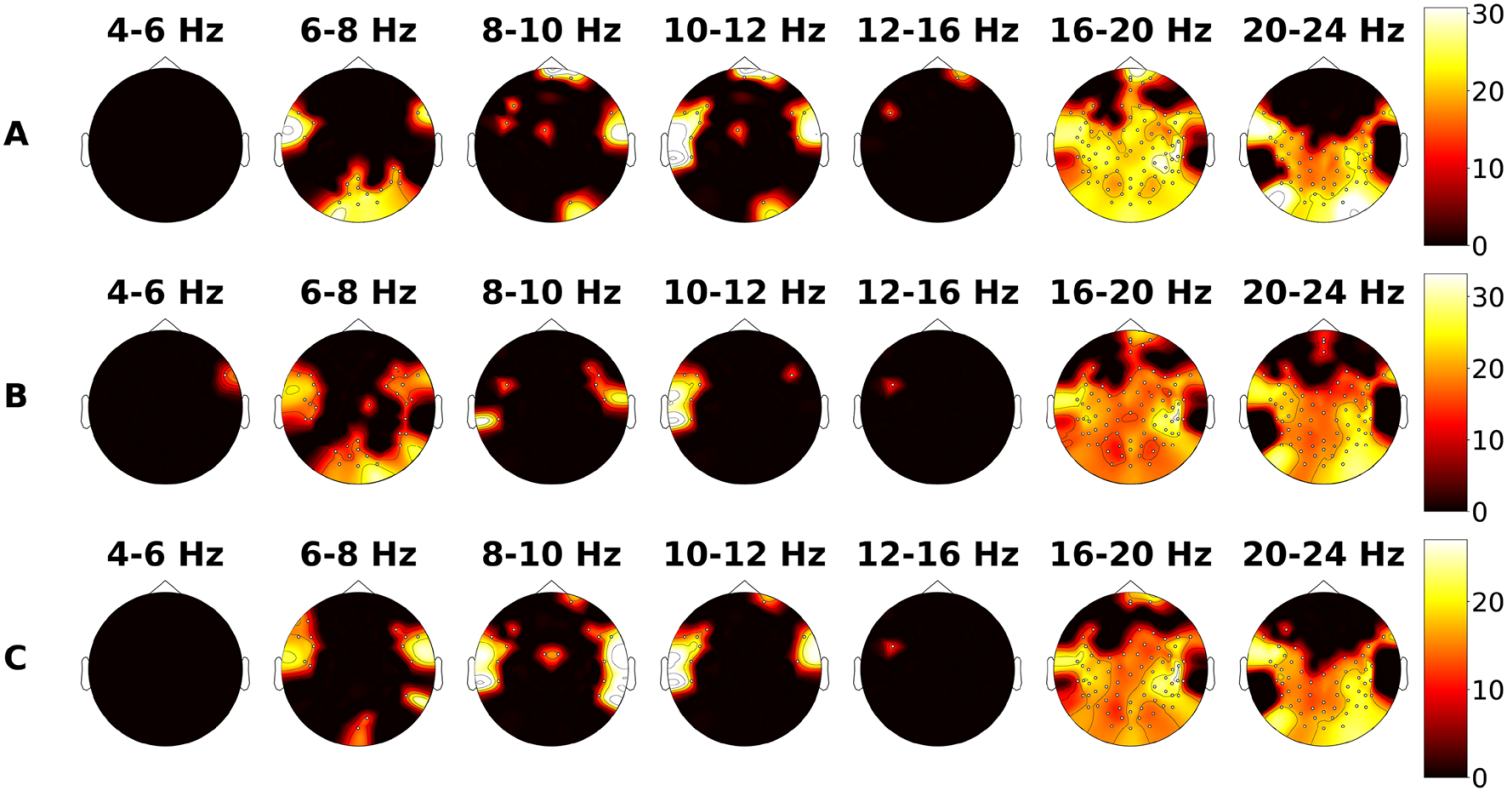
The topographic distribution of differences in EEG PSD between the experts in mathematics (M group) and experts in humanities (H group) during the solving of three different types of tasks is shown in panels (**A**–**C**) for verbal tasks, arithmetic tasks, and logical tasks, respectively. The color bar represents the T-statistic.

Using a two-sided permutation cluster test, we identified significant differences in power spectral density (PSD) of EEG between two groups (M and H) in several clusters. The results using T-statistic for each type of task are presented in Fig. 1. The significant clusters indicate that the PSD of EEG under these locations of sensors was either higher or lower in M group compared to H group.

In the lower theta range (4–6 Hz), significant clusters of differences in EEG between M and H group were found only for EEG during arithmetic task solving in the posterior lateral frontal region. In the upper theta range (6–8 Hz), significant clusters were found for all types of tasks in the occipital region and bilaterally in the junction of temporal and frontal regions.

Significant clusters were found in the lower alpha band (8–10 Hz) in the central and frontal regions for EEG during the solving of verbal and logical tasks. Moreover, bilaterally in the temporal region, significant clusters were found for all types of tasks except for verbal, where they were only found in the right temporal region. Similarly, in the upper alpha band (10–12 Hz), significant clusters were found in the frontal region for EEG during the solving of verbal and logical tasks. Furthermore, bilaterally in the temporal region, significant clusters were found for all types of tasks except for arithmetic, where they were only found in the left temporal region.

In the lower beta band (12–16 Hz), significant clusters were found in the left posterior frontal region for all three types of tasks. In addition, significant clusters were located in the frontal region for verbal tasks. In the middle and upper beta range (16–20 Hz; 20–24 Hz), significant clusters for all types of tasks were found almost throughout the entire head surface, except for the entire frontal region in both bands, the right temporal region in the middle beta band (16–20 Hz), and both sides of the temporal region in the upper beta band (20–24 Hz).

### Subject-independent classification of the group type

**Figure 2:**
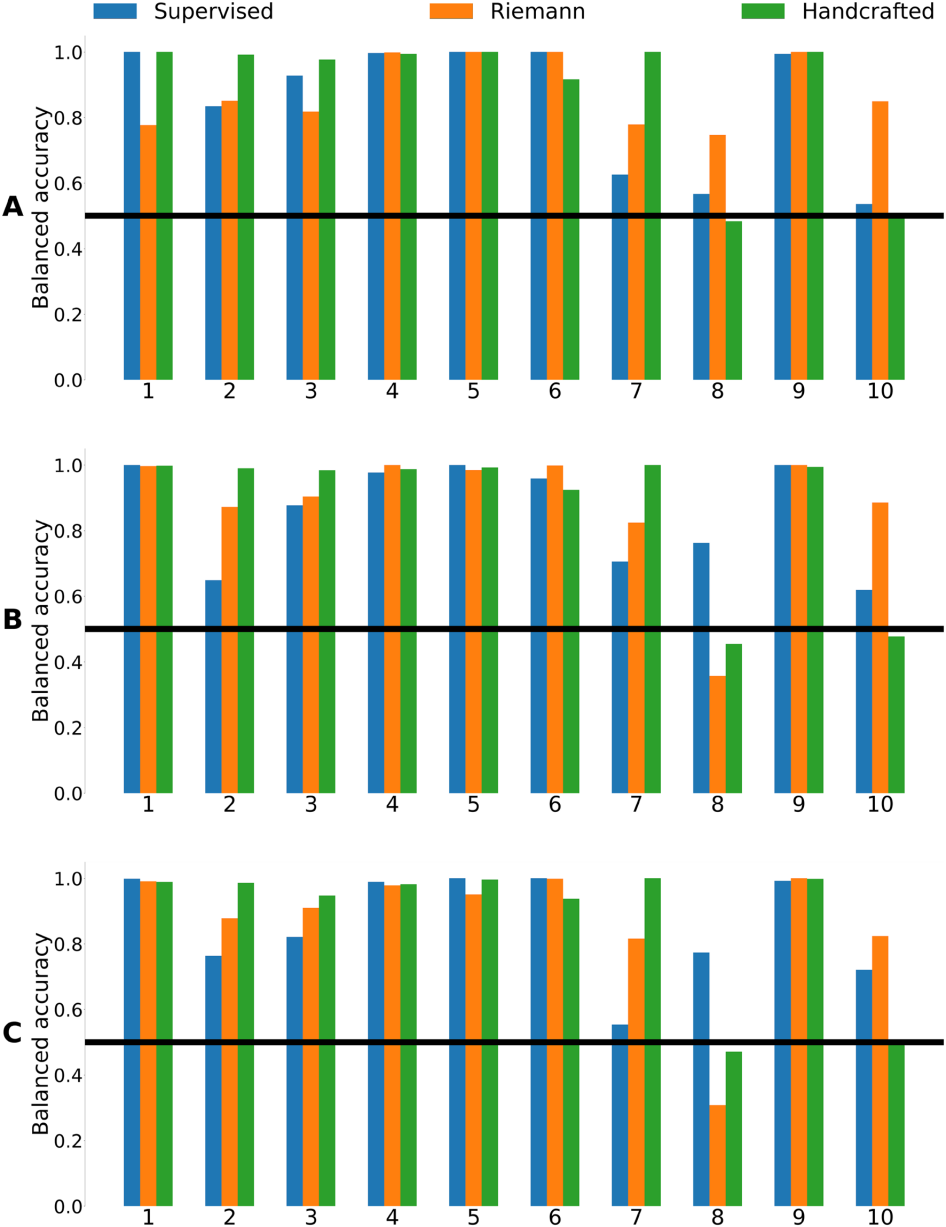
Balanced accuracy for cross-subject classification of the group type using different pipelines and types of tasks: (**A**) verbal, (**B**) arithmetic and (**C**) logical tasks.

**Figure 3:**
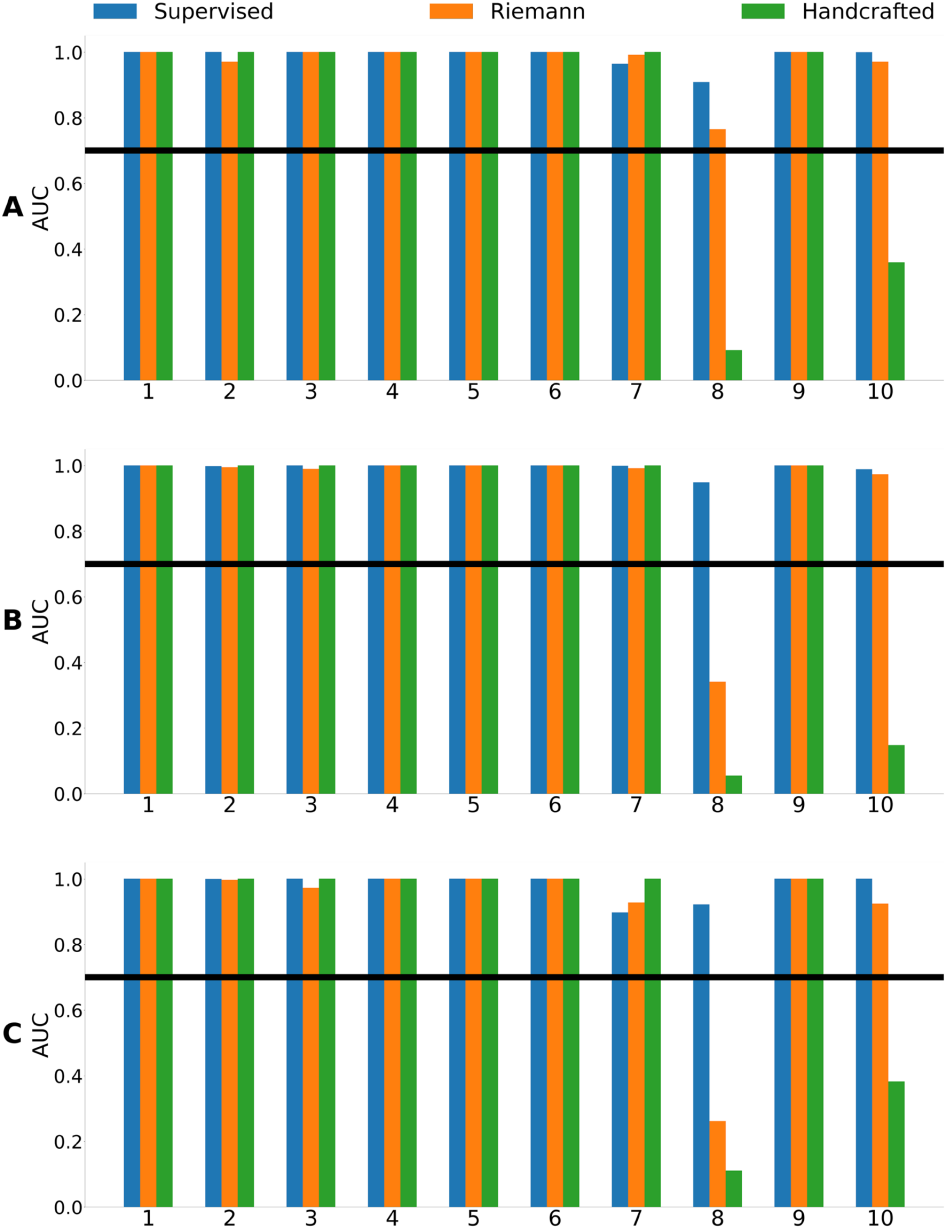
AUC for cross-subject classification of the group type using different pipelines and types of tasks: (**A**) verbal, (**B**) arithmetic and (**C**) logical tasks.

The BA and AUC metrics for cross-subject classification of the group type for each fold are presented in Figs. 2 and 3. Mean metrics are presented in in Table 1. There were no significant differences in performance between the models as assessed by the Wilcoxon signed-rank test.

**Table 1:** Classification performance as indicated by accuracy and ROC area under the curve (AUC).

Pipeline	Metric	Verbal	Arithmetic	Logical
Supervised	Accuracy	$0.84\pm 0.034$	${\varvec{0.88\pm 0.010}}$	${\varvec{0.89\pm 0.039}}$
	AUC	$0.99\pm 0.001$	${\varvec{0.97\pm 0.005}}$	$0.85\pm 0.099$
Riemannian	Accuracy	$0.85\pm 0.022$	${\varvec{0.88\pm 0.034}}$	$0.88\pm 0.043$
	AUC	${\varvec{0.99\pm 0.0002}}$	$0.93\pm 0.038$	$0.82\pm 0.129$
Handcrafted	Accuracy	${\varvec{0.86\pm 0.022}}$	$0.86\pm 0.039$	$0.88\pm 0.039$
	AUC	$0.98\pm 0.001$	$0.91\pm 0.047$	${\varvec{0.85\pm 0.094}}$

Best accuracy and AUC metrics are indicated by bold.

**Figure 4:**
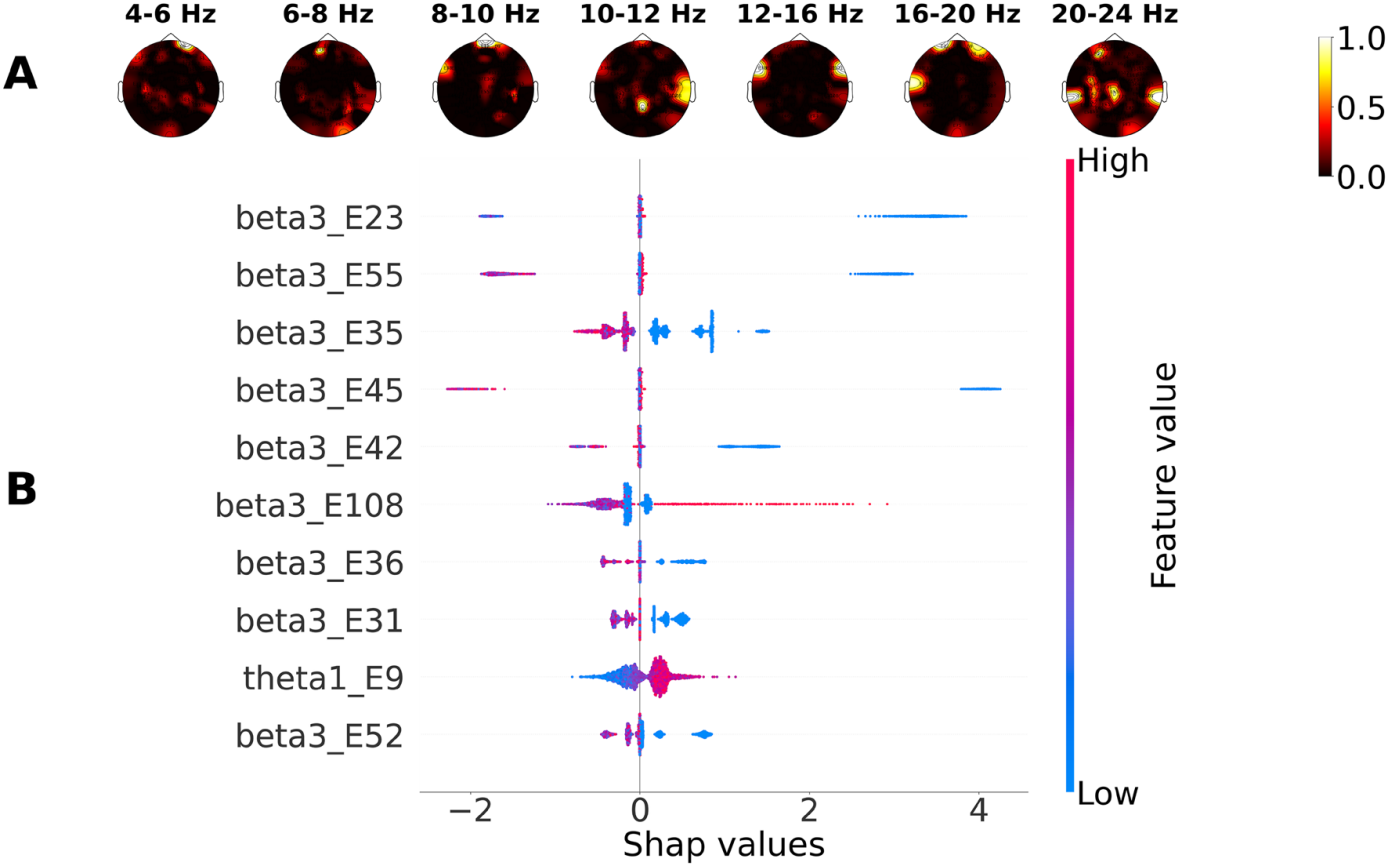
SHAP values for LightGBM classifying the subject group based on EEG during the solving of verbal tasks: (**A**) mean SHAP values, (**B**) top 10 most important features and explanation of their effect on the probability that a subject belongs to the M group.

**Figure 5:**
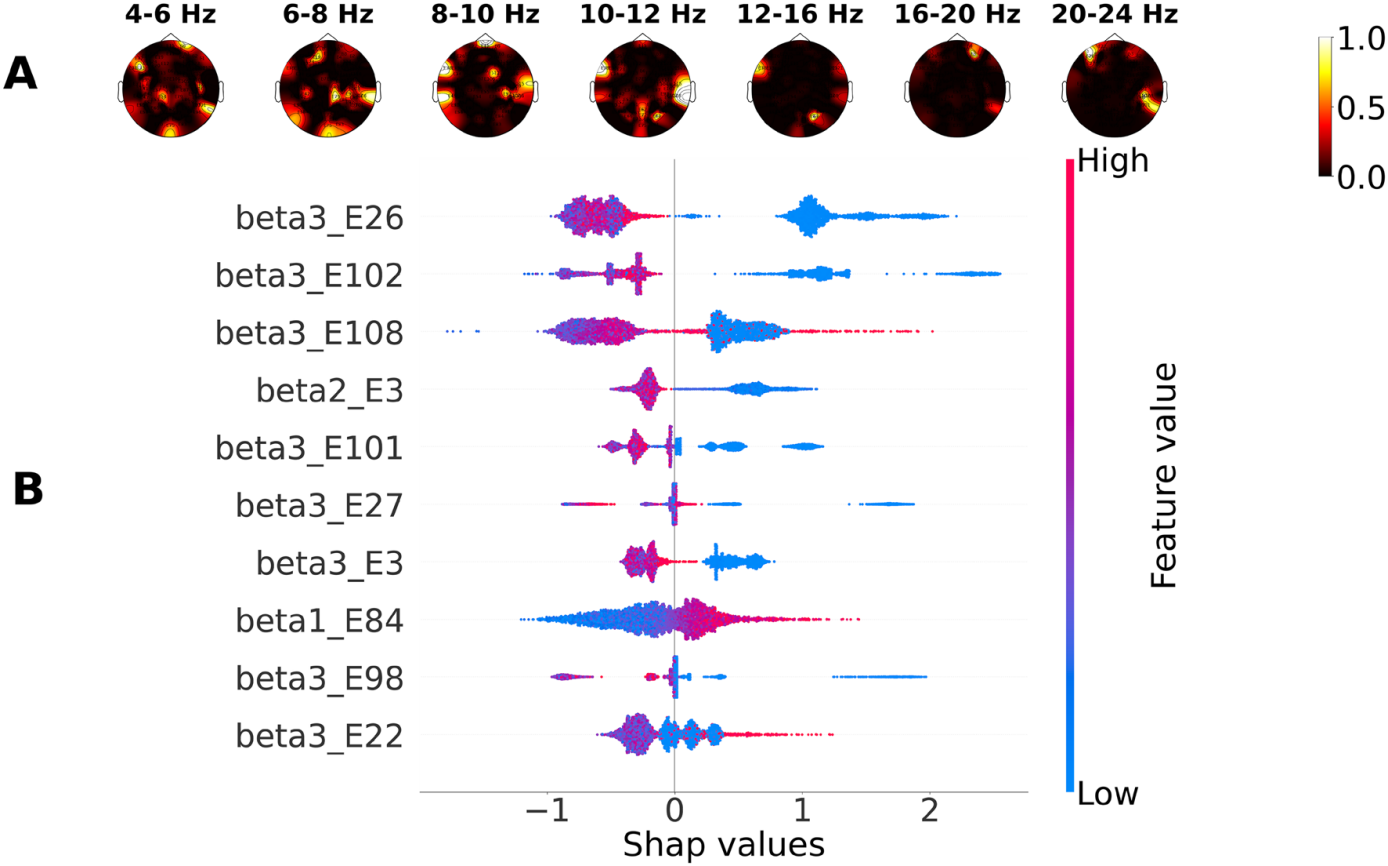
SHAP values for LightGBM classifying the subject group based on EEG during arithmetic task solving: (**A**) Mean SHAP values, (**B**) Ten most important features and an explanation of their effect on the probability that the subject belongs to the M group. The colorbar represents the feature value.

**Figure 6:**
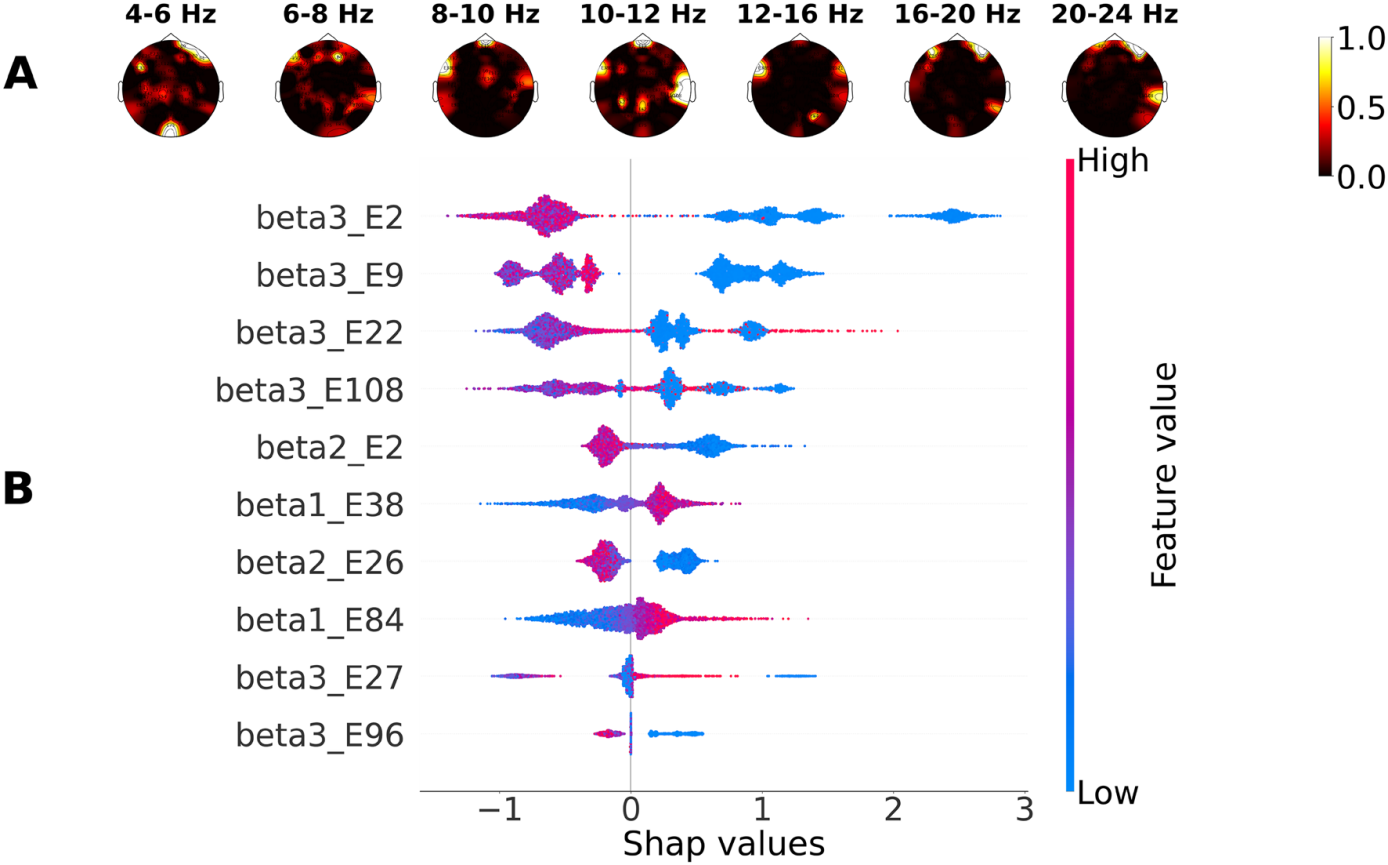
SHAP values for LightGBM classifying the subject group based on EEG during logical task solving: (**A**) mean SHAP values, (**B**) 10 most important features, and their corresponding effect on the probability that the subject belongs to the M group. Color bar represents the feature value.

SHAP values for LightGBM and different types of tasks are presented in Figs .4, 5 and 6.

Significant features for correct classification based on the mean SHAP values were scattered across all frequency ranges, mainly in the temporal, frontal, and occipital areas, predominantly in the right hemisphere. An association of classification features with both types of mathematical tasks was observed for PSD of 4–6 Hz in the central occipital region and bilateral junctions of the temporal and parietal regions, more prominently for logical tasks, and for PSD of 8–10 Hz in the central region and bilateral temporal regions.

Based on explained SHAP values for LightGBM, the ten most important features were located as follows:

- For verbal tasks in the low theta range (4–6 Hz), above the right prefrontal electrode; in the low beta range (12–16 Hz), above the bilateral temporal electrodes; and in the high beta range (20–24 Hz), above the left frontal area, above the junction of central and left temporal regions, above the right temporal electrode, and central occipital electrodes.
- For arithmetic tasks, predominantly in the high beta range (20–24 Hz) above bilateral frontal and the right temporal-parietal region; in the low beta range (12–16 Hz) above the left temporal and right occipital electrodes; and in the middle beta range (16–20 Hz) above the right frontal electrodes.
- For logical tasks, predominantly in the high beta range (20–24 Hz) on bilateral frontal areas, above the right temporal and parietal electrodes; additionally in the low beta range (12–16 Hz) above the left temporal and right occipital electrodes; and in the middle beta range (16–20 Hz) above bilateral frontal electrodes.

## Discussion

This study illustrates the feasibility of identifying the educational background of healthy individuals using ML algorithms and EEG data during cognitive tasks. The classification accuracy (BA) was consistently above chance for all three applied pipelines (Supervised, Riemannian, Handcrafted) across verbal, arithmetical, and logical tasks.

Most BCI studies, even today, are sensitive to individual differences^33^, due to both high individual variability of human EEG and behavioral strategies^18^. Cross-individual classification of cognitive skills has high practical value for the creation of passive BCIs^33,34^ that can be utilized in the process of education, psychological assessment and medical diagnosis. However, due to the non-stationarity and subject specificity of EEG time-series, training robust models is a challenging task. Nevertheless, ML methods can be applied to subject-independent classification. A cross-subject classifier with CSP features was developed to recognize two mental states, happiness and the imagination of movement, with an accuracy of 75.30% ^35^. In^17^, generative adversarial networks and transfer learning were used for subject-independent fatigue classification with high accuracy, up to 91.63%. The most successful utilizing transfer learning was reported in cross-subject classification of motor-imagery tasks^36,37^. Numerous studies have been devoted to the classification or identification of group differences between healthy individuals and individuals with neurological diseases, such as Alzheimer’s^38^, chronic pain^39^, or autism^40^. However, very few studies have tested the classification of healthy individuals by their cognitive performance because it involves complex relationships in sensor-frequency space and is difficult for statistical or machine learning inferencing^24^.

In this study, we applied the cluster permutation test due to its ability to localize effects in high-dimensional and structural data, and three different ML pipelines: CSP and Riemannian vectorization with linear model and power features with LightGBM for the classification of subjects by their educational background—mathematicians versus specialists in humanities. The permutation test demonstrated the presence of a significant statistical difference between the two groups, mainly in the upper theta (6–8 Hz) and in the two upper beta ranges (16–20 Hz), (20–24 Hz). It is worth noting that the cluster permutation test should be interpreted carefully because it does not control the frequency of false positives at the channel, frequency, or time levels, and it imposes constraints on probabilistic statements about effects at these levels^41^.

To measure the quality of the generalization ability of the classification, we used two metrics: BA and ROC-AUC. AUC is considered not only a quality indicator resistant to the influence of various subjective factors but also not sensitive to non-uniform distribution in the data^42^. With all ML pipelines, we obtained statistically significant results. In the absence of a statistical difference between all pipeline results, preference in neuroscience studies should be given to interpretable machine learning methods or, no less importantly, physiologically interpretable features.

However, our results show that the most important features for classification of the expertise type (M or H) could be scattered across all frequency bands, making the interpretation quite difficult. To overcome this problem, we used the SHAP method and obtained the 10 features with the highest SHAP values implying the feature significance in classification for each type of task independently. For mathematical and logical tasks, the most important features, allowing to recognize the expertise type, prevailed mainly in the beta range above the frontal lobe and on the right parietal temporal area in individual EEG data from the M group. Beta synchronizations are able to provide neural communication over distinct brain regions, primarily due to interactions between the posterior parietal and prefrontal cortex^43^. Beta rhythm in the frontal cortex is closely related to the processes of executive control of working memory^44^. Beta phase synchronization was previously associated with a wide range of cognitive processes, including attention control^45,46^, guided visual search, and free choice^43^. It has been shown that during the perception and calculation of numbers, the right parietal regions are systematically activated^23,47^. The temporal-occipital regions are involved in the process of visual processing of symbolic numerical information^48^. The coherence in activation of bilateral temporal lobes was also associated with cognitive processes related to academic performance in mathematics^19^. It has also been reported that the right temporal areas are involved in the neural efficiency of the mathematically gifted brain^49^. Thus, our results confirm that interpretation methods in ML are capable of reproducing group statistical comparison of EEG patterns.

To conclude, our results reinforce the use of ML in the detection of individual cognitive features by EEG, as all three applied ML pipelines allowed us to distinguish mathematical proficiency depending on experience in learning with different trade-offs between performance and explainability, without transfer learning.

As limitations of the study, it should be mentioned that we utilized pre-defined frequency bands based on extensive prior research data to facilitate cross-subject classification and ensure the interpretability of machine learning results. While data-driven methods could be employed to identify EEG frequency boundaries, this would potentially lead to more precise classification of individual cognitive traits in future EEG data analyses. Additionally, we inferred comparable intellectual levels between the two groups (M and H) based solely on participants’ academic degrees. Finally, the relatively moderate sample size represents a significant limitation for findings. The tentative conclusion suggests a connection between math proficiency and rhythmic brain activity, supported by significant differences detected between M and H groups using the cluster permutation test and the positive outcomes of machine learning models with 10 validation folds. However, a more robust inference would require a study on a larger sample size.

## Methods

### Participants

Twenty-seven healthy right-handed volunteers participated in the study. Participants were recruited via social networks after they filled an online screening form for neurological and psychiatric disorders. Data from one individual were excluded from the analysis due to extensive EEG artifacts. The final study sample consisted of 26 participants (age 25.7 ± 4.49, range 19-38; 12 females, 14 males) divided into two groups: M group (12 students or specialists with professional math education and experience), and H group (14 students or specialists in humanities). The principle of dividing individuals into groups based on education was as follows: the participants of the M group were either students of at least the third year of universities in mathematical specialties or working alumni of these universities. The same applies to the participants of the H group, but in humanitarian specialties (history, philology, law). This study was conducted in accordance with the Declaration of Helsinki and approved by the Ethics Committee of the Institute of Higher Nervous Activity and Neurophysiology of the Russian Academy of Sciences (protocol No3 from 24 August 2017). Volunteers have given written informed consent to their participation in the study, after the procedure was explained to them.

### Experimental design

Participants were comfortably seated in a sound-shielded room, 1 meter away from a 19-inch square monitor. During EEG recording, participants were presented with tasks and were asked to mentally solve them, giving equal priority to accuracy and the shortest solving time. The tasks were presented in light gray in the center of a black screen, with the sizes of letters and digits being equal for all tasks. We presented three types of tasks (Table 2) in a pseudorandom order: 60 verbal, 60 arithmetic, and 60 logical tasks. The trial sequence consisted of task instructions (2 s), a fixation cross (0.5 s), the task (< 40 s), and a black screen during the participant’s response (4 s). Tasks were presented with a limited duration of 40 s. Participants clicked the left PC mouse button when they were ready to provide an answer. If no response was given within 40 s, the task was considered unsolved, disappeared from the screen, and the next trial began. The decision time (DT) was calculated between the task onset and the participant’s response. Additional long breaks were provided every 20-30 minutes or upon the participant’s request. The entire experiment typically lasted 2-2.5 hours.

**Table 2:** Types and examples of tasks.

Type of task	Description	Example	Number
Verbal task	Anagram of 5-letter words	btela - table	30
	Anagram of 6-letter words	wnesra - answer	30
Arithmetic task	Addition	37+41+29+8	12
	Subtraction	9945-2417	12
	Multiplication	41*9-23*3	24
	Fraction	34/6-27/12	12
Logical task	Extension of integer sequence	2; 6; 11; 33; 38 ...	60

### EEG recording and preprocessing

The EEG was recorded using a 128-channel Geodesic Sensor Nets (Electrical Geodesics, Inc (EGI), Eugene, OR, USA) system based on the 10–10 electrode montage. The recordings were band-pass filtered with a 0.1 Hz–70 Hz analog filter, notch filtered at 50 Hz, and sampled at 1000 Hz with online re-referencing to the average using Net Station software. Impedance was kept below 50 K$\Omega$.

All preprocessing was done using MNE software^50^. We excluded 46 “skirt channels” (defined as channels with EGI polar coordinate r > 0.5) near the periphery of the EEG net that are particularly sensitive to noise and muscle artifact. The remaining 83 channels retained for analysis is provided in the supplementary materials and labeled with their EGI channel number.The number of discarded channels was consistent across conditions and participants.

Remaining data were downsampled to 250 Hz. EOG artifacts were removed using automatic ICA in MNE. ICA components were found on high-passed filtered at 1 Hz signal and applied to unfiltered signal. The EEG data were analyzed in epochs of 2 s without overlap starting 5 s after the presentation of each task. As we were more interested in the recognition of mental operations during task solving rather than in the visual perception of the tasks, we assumed that during the first 5 s of cognitive task presentation, EEG could reflect visual retrieval and early (stimulus-driven bottom-up) cognitive stages related to semantic and lexical numerical representations of the stimuli. EEG, accompanied by the visual perception of complex visual stimuli such as math and verbal tasks, has a strong impact on eye-movement activity. Epochs with low signal-to-noise ratio were rejected using minimum and maximum peak-to-peak amplitudes. 94.1 ± 2.2 % of epochs remained after preprocessing. Following the epoch rejection, there were no statistically significant differences in the number of epochs between the groups (M and H) and conditions.

### Analysis of behavioral performance

A mixed-model ANOVA (GROUP, TASK) was applied to compare results on behavioral performance between the groups and 3 types of the tasks. As the distribution of behavioral variables deviated from normal we applied cluster based permutation tests with Spearman coefficient to perform a correlation analysis of EEG patterns and behavioral performance. We examined the mean PSD value of 96-channel data for each frequency range in correlation with behavioral results.

### Classification methods

We performed cross-subject group classification to recognize the group type to which the individual EEG data belonged. Additionally, we compared the performance of three pipelines: supervised and unsupervised projections with Logistic Regression and handcrafted power features with LightGBM.

#### Feature space

The frequency bands used for feature estimation were as follows: $\theta$1 (theta1): 4–6 Hz, $\theta$2 (theta2): 6–8 Hz, $\alpha$1 (alpha1): 8–10 Hz, $\alpha$2 (alpha2): 10–12 Hz, $\beta$1 (beta1): 12–16 Hz, $\beta$2 (beta2): 16–20 Hz, $\beta$3 (beta3): 20–24 Hz. For the first two pipelines, we decomposed EEG into these bands using a set of filter banks with Butterworth Bandpass filters, and for each epoch in each band, the covariance spatial matrix was estimated. The feature space before projection and vectorization was ${\varvec{X}}\in {{\mathbb {R}}}^{K\times N\times N}$, where *K* is the number of frequency bands and *N* is the number of EEG channels.

For the third pipeline with handcrafted features, power spectral density (PSD) for each epoch and frequency band was estimated using the multitaper method^51^ that calculates spectral density for orthogonal tapers and then averages them together for each channel. Relative power, equal to the power in each frequency band divided by the total power, was used to form the feature space for classification. Thus, the feature space in this pipeline was ${\varvec{X}}\in {{\mathbb {R}}}^{N\times K}$, where *K* is the number of frequency bands and *N* is the number of EEG channels.

#### Supervised spatial projection with log-vectorization

In the space of symmetric positive-definite (SPD) matrices^29^, the covariance matrices of the CSP filtered signal will take the form of (1), where ${\varvec{W}}\in {{\mathbb {R}}}^{N\times J}$ and $J \le N$ is the number of CSP filters sorted by decreasing eigenvalues. Thus, the feature vector will take the form of (2) with the number of components equal to *J*, where ${\varvec{\Sigma _{1}}}$ is the mean covariance matrix of the first class.

1 $$\begin{aligned} {\varvec{\Sigma _{{\varvec{Z}}_{i}}}}= & {} {\varvec{W^{T} \Sigma _{{\varvec{X}}_{i}} W}} \end{aligned}$$

2 $$\begin{aligned} {\varvec{F}}_{i}= & {} \begin{bmatrix} \log ({\varvec{\Sigma _{Z_{i}}}}\left[ 1,1\right] ) - \log ({\varvec{\Sigma _{1}}}\left[ 1,1\right] ) \\ \vdots \\ \log ({\varvec{\Sigma _{Z_{i}}}}\left[ J,J\right] ) - \log ({\varvec{\Sigma _{1}}}\left[ J,J\right] ) \end{bmatrix} \end{aligned}$$

#### Unsupervised spatial projection with Riemann vectorization

In the space of SPD matrices^52^, the covariance matrix of the signal projected with Principal Component Analysis (PCA) will take the form of (3), where ${\varvec{W}}\in {{\mathbb {R}}}^{N\times J}$ and $J \le N$ is the number of PCA components sorted by decreasing eigenvalues. Therefore, the minimal representation of a matrix in the Riemannian space will take the form of (4), where ${\varvec{F}}{i}$ is a feature vector with the number of components equal to $J*(J+1)/2$, and $\overline{{\varvec{\Sigma }}}{Z}^{-1/2}$ is the mean of (3) matrices according to the Riemannian metric.

3 $$\begin{aligned} {\varvec{\Sigma }}_{{Z}_{i}}= & {} {\varvec{W}}_{UNSUP}^{T} {\varvec{\Sigma }}_{{X}_{i}}{\varvec{W}}_{UNSUP} \end{aligned}$$

4 $$\begin{aligned} {\varvec{F}}_{i}= & {} {Upper}(\log (\overline{{\varvec{\Sigma }}}_{Z}^{-1/2}{\varvec{\Sigma }}_{{Z}_{i}}\overline{{\varvec{\Sigma }}}_{Z}^{-1/2})) \end{aligned}$$

#### Logistic regression

Logistic regression is a linear model employed to estimate the likelihood of a specific class, and it can serve as a supervised binary classification algorithm. To avoid overfitting, logistic regression was trained with $L_2$ regularization, and the regularization parameter for $L_2$ was specified within the range of $\left[ 1e^{-10},1e^{9}\right]$.

#### LightGBM

LightGBM is a gradient boosting framework that uses a decision tree algorithm with leaf-wise split, and is known for its high performance. To optimize its performance, a cross-validation grid search was used to tune its hyperparameters.

#### Models training and validation

To perform subject-independent classification of the participant group (M versus H), EEG epochs were labeled according to the group to which the subject belonged. For testing, epochs from two randomly selected participants from different groups were chosen, while epochs from two randomly selected participants (one from each group) were chosen for validation. The remaining participants epochs were used for training. This process was repeated ten times, resulting in ten different folds for each type of task.

### Classification metrics

Balanced accuracy (BA), receiver operating characteristic (ROC) curve and area under the curve (AUC) were used to assess the performance of the models.

### Feature explainability

#### Filters and patterns

One advantage of linear models is their interpretability, which allows for the identification of the strength and direction of specific effects in the features^30^.

In classification tasks, the backward model transforms the feature space ${\varvec{X}}{i} \in {{\mathbb {R}}}^{N\times 1}$ into a new representation that maximizes the discriminability between the two classes using the filter ${\varvec{W}}\in {{\mathbb {R}}}^{N\times 1}$ as shown in Eq. (5). On the other hand, the forward models in Eq. (6) describe sample generation as a multiplication of the activation pattern ${\varvec{A}} \in {{\mathbb {R}}}^{N\times 1}$ by the factor $s_{i}$. The activation factor can be obtained using Eq. (7), where the covariance matrix is ${\varvec{\Sigma _X}} = \mathbb E\left[ {\varvec{X}}{i},{\varvec{X}}{i}^{T}\right] _{i}$.

5 $$\begin{aligned} {{\varvec{W}}}^{T}{\varvec{X_{i}}}= & {} {\hat{s}}_{i} \end{aligned}$$

6 $$\begin{aligned} {\varvec{x_{i}}}= & {} s_{i}{\varvec{A}}+{{\varvec{\varepsilon }}_{i}} \end{aligned}$$

7 $$\begin{aligned} {\varvec{A}}= & {} {\varvec{\Sigma _{{\varvec{X}}} W \Sigma _{\hat{{\varvec{s}}}}^{-1}}} = {\varvec{\Sigma _{{\varvec{X}}} W}} = Cov\left[ {\varvec{X}}_{i}, s_i\right] \end{aligned}$$

When using CSP, the feature space takes the form of ${\varvec{X}}_{i} \in {{\mathbb {R}}}^{NK\times 1}$ and the filter takes the form of ${\varvec{W}} \in {{\mathbb {R}}}^{NK\times 1}$. This filter has full column rank, which is proven by using the Sylvester rank inequality in Eq. (8).

8 $$\begin{aligned} rank({\varvec{W_{1}}}) + rank({\varvec{W_{2}}}) - M \le rank({\varvec{W}}) \le min(rank({\varvec{W_{1}}}),rank({\varvec{W_{2}}})) \end{aligned}$$

#### SHapley Additive exPlanations

For examining the feature importance in the case of LightGBM, we estimated the SHapley Additive exPlanations (SHAP) values^32^. SHAP values assign an importance value to each feature in a model. Features with positive SHAP values positively impact the prediction, while those with negative values have a negative impact. The magnitude is a measure of how strong the effect is.

### Statistical comparison of EEG patterns between tasks and groups

A cluster-based permutation test^53^ was used to investigate differences in EEG power spectral density (PSD) between groups. A two-sided T-statistic with a threshold of 6 was applied and corrected for multiple comparisons using N=1024 permutations. Cluster-level correction based on spatial adjacency was also performed.

### Supplementary Information

Supplementary Information.

## Data Availability

Anonymized data are available upon request from the corresponding author.
