# Supplementary material for: Detecting cognitive traits and occupational proficiency using EEG and statistical inference
Source: Sci Rep. 2024 Mar 7;14:5605. doi: 10.1038/s41598-024-55163-w (PMC10920811; doi:10.1038/s41598-024-55163-w)

Detecting Cognitive Traits and Occupational Proficiency Using EEG and Statistical Inference

Ilya Mikheev 1,*, Helen Steiner 2 and Olga Martynova 2,3

1 HSE University, Department of Psychology, Moscow, 101000, Russia
2 Institute of Higher Nervous Activity and Neurophysiology of the Russian Academy of Sciences, Moscow, 117485, Russia
3 HSE University, Centre for Cognition and Decision Making, Moscow, 101000, Russia
* imikheev@hse.ru

1. List of discarded channels

'E8', 'E14', 'E17', 'E21', 'E25', 'E43', 'E44','E48', 'E49', 'E56', 'E57', 'E63', 'E64', 'E65', 'E68', 'E69', 'E73', 'E74', 'E81', 'E82', 'E88', 'E89', 'E90', 'E94', 'E95', 'E99', 'E100', 'E107', 'E113', 'E114', 'E119', 'E120', 'E125', 'E126', 'E127', ‘E128'

2. ROC plots, AUCs, filters and patterns of CSP corresponding to the largest eigenvalue for each type of task

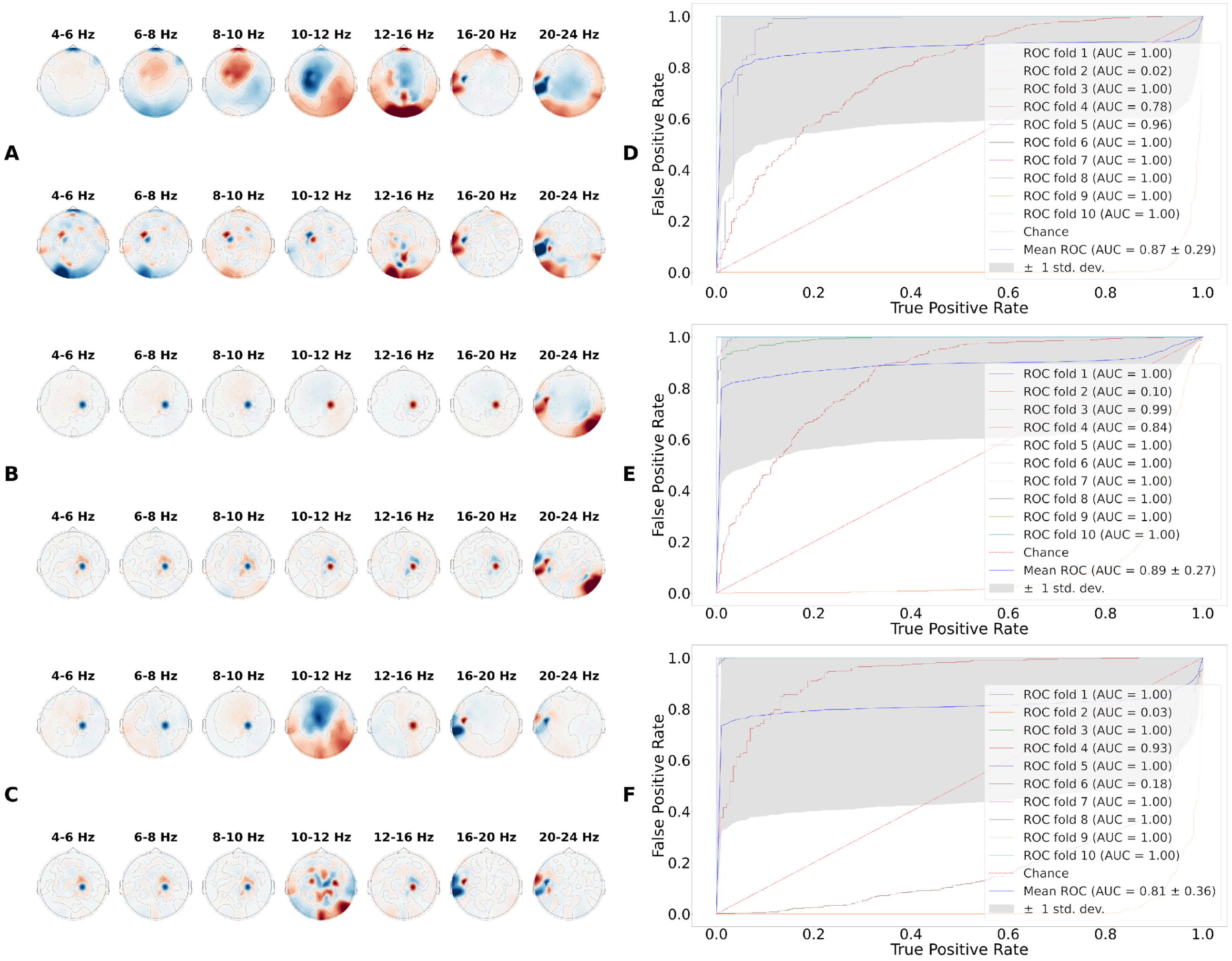

Supplement: Supplementary file 1 — Supplementary Information. [file 41598_2024_55163_MOESM1_ESM.pdf]
